# Urine

**Published:** 1861-10

**Authors:** 


### Urine.

*Urine as a means of Establishing the Diagnosis and Treatment of Diseases.*—Dr. F. Simon gives the following valuable communication: Among the earlier investigators who paid attention to the urine, though very partially, I may mention Brandt, Kunkel, Boyle, and Bellini; Boerhaave, however, attempted an analysis of the urine, which, considering the time, was extremely good. Scheele's discovery of uric acid, and Cruickshanks' of urea, contributed essentially to a more correct knowledge of this secretion. The latter surgeon had already examined the urine in several diseases, especially in diabetes and dropsies.

At the commencement of the present century it was chiefly Berzelius and Prout who made the urine the subject of extended inquiries; Berzelius demonstrated the existence of lactic acid, which, by the earlier chemists had been considered to be acetic acid; the analysis communicated by Berzelius, in 1809, of the composition of the urine, has been, till within the last few years, the only correct examination of the same; Prout has continued his inquiries up to the latest period.

Of the more recent works on the constitution of the urine, those by Lecanu are the most prominent; within the last years, Becquerel, Lehmann, and Simon, have employed themselves with examinations of the urine in the healthy and morbid state.

Several constituents of the urine, both in the state of health and disease, are very accurately known—as uric aid, urea, lactic acid—the salts and the sugar of the urine; of others, probably not less important, we have a very imperfect knowledge, as of extractive and coloring matters.

Regarding the quantitative composition of the urine, which is rather changeable, numerous investigations have been made by the above-named chemists. Lecanu also investigated the varieties which may be shown in healthy urine, according to age and sex.

The quantity of urine passed in the twenty-four hours, and its color, are frequently of importance.

A diminished quantity of the urine passed in twenty-four hours, is, under circumstances, a sign particularly of acute diseases; an excessive increase of the urine, if permanent, is oftentimes indicative of serious diseases.

A dark-colored, flaming, or fiery-red urine commonly indicates an inflammatory affection.

A dark brown red is generally observed in typhus.

But the urine may also be colored blood-red or brown-red by bile-pigment, which is easily detected by its reaction with nitric acid; the latter constantly indicates an affection of the liver.

A blood-red urine commonly contains blood; there is then for the most part found in it a sediment of blood-corpuscles, which are recognized with the microscope; but should a little blood be contained in the urine, and this in a state of solution, it may be discovered by adding nitric acid, which occasions a precipitation of coagulated albumen colored red by hematine. This bloody urine indicates a bleeding in the kidneys, bladder, or, in the case of women, of the uterus.

Blood flowing from the urethra comes in drops. If the blood is discharged in masses after clear urine, it comes from the bladder, and in that case it often stops up the passage from the bladder by coagulation; if the blood is distributed through the urine, partly dissolved, and not in very large quantity, it comes from the kidneys; if it be dark, and mixed with mucus and pus, it owes its origin to an ulcer. The presence of stone-colic shows that the blood has been poured out during the descent of a renal calculus.

Blue urine has been observed, though not frequently; in the majority of cases it probably owes its origin to the use of certain medicines.

Black urine has likewise been observed; the connection, however, is not yet known between the coloring matter and the morbid process.

Greenish urine indicates, according to Prout, an oxalic acid diathesis; sediment of oqalate of lime form, or mulberry calculi pass away.

A urine, which is pale-colored, and has a bias to green, frequently indicates the presence of albumen, which is readily detected by heating to boiling or by nitric acid. In this case the urine is not perfectly clear, but slightly opalescent; its quantity may be increased, diminished, or natural.

The oxalic acid diathesis of the urine indicates, according to Prout, functional disturbances in the chylopoietic system.

Albuminuria ordinarily indicates dropsy and an affection of the kidneys.

The reaction of the urine is important for the physician.

Natural urine, it is well known, has an acid reaction: the quantity of free acids in the urine and the intensity of the reaction may increase to an extraordinary degree in diseases—more particularly in rheumatism, gout, in disturbances of the digestive organs, and in certain stages of typhus; to judge correctly of the intensity of the acid reaction, reference must be had to the quantity of the urine; the greater or less acid reaction is known by the effect of the urine on litmus paper of a weak blue color, which becomes colored so much the more rapidly and the more deeply reddened, the greater the acid contents of the urine are.

Urine with a neutral reaction commonly forms the transition from the acid to the alkaline reaction, and *vice versa.*

The alkaline reaction of the urine is of great importance to the physician; it commonly depends on carbonate of ammonia, the presence of which is recognized by the odor, and the white cloud, which a glass rod develops when moistened with an acid salt and brought near to it.

The urine also may have an alkaline reaction through its containing carbonate of soda, which salt finds its way into the urine by the long-continued use of carbonate or bicarbonate of soda with vegetable acids.

The urine, alkaline by carbonate of ammonia, is but seldom evacuated in this state from the bladder; during its discharge it is commonly neutral, and becomes alkaline only in a shorter or longer time after; badly-cleaned vessels may moreover contribute much to this, a circumstance which ought to be taken into account.

Urine which, already on voiding it, has an ammoniacal reaction, and has also a very bad smell, indicates always a serious affection of the nervous system, and especially of the spinal cord.

In certain unfavorable stages of tabes dorsalis, phthisis of the spinal cord, paralysis of the lower extremities and of the bladder, the voiding ammoniacal urine is ever an unfavorable sign; in other affections of the nervous system, also, as in typhus, ammoniacal urine is observed, which, however, assumes this reaction in the majority of cases not till after it has stood for some time.

In typhus the reaction of the urine may be of importance for the prognosis, when the urine, after it was observed to have an acid reaction through one, two, or three periods of seven days, is finally found to be neutral, and then to have an ammoniacal odor and reaction; when this reaction lasts for several days, probably during one entire period of seven days, and then again passes into the acid, this seems in most cases to indicate a favorable termination to the disease.

The urine having an ammoniacal reaction in typhus has usually a dirty, turbid, yellow-brown or red-brown appearance, and forms sediments which disappear in a great measure on the addition of free acids; also in catarrhus vesicæ, or in phthisis vesicæ, the urine becomes ammoniacal in a very short time after being voided; the large quantity of vescal mucus or pus indicates this affection; finally, the formation of urinary concretions, consisting of earthy phosphates, is in part occasioned by the neutral or alkaline reaction of the urine; the urine voided in this urinary affection, is not so dark as the urine in typhus, and commonly forms sediments of phosphate of lime, and of ammonio-magnesian phosphate.

If the vesical calculus exercise an irritating influence on the parietes of the bladder, a great quantity of vesical mucus is commonly mixed with the urine.

The specific gravity of the urine, though by itself it possesses no great diagnostic value, as it depends on the variable quantity of water in the urine, may, however, claim the attention of the physician under certain circumstances.

The clearer and the more like water the urine appears, the less is its specific gravity; the deeper and darker-colored, the higher the specific gravity.

This general law may admit of an exception in one case, namely, in diabetes mellitus; in this disease a urine is voided either normal or pale, seldom deeply-colored, the high specific gravity of which (1020-1060) is in contradiction to the color; this high specific gravity imperatively requires a more strict examination of the urine.

More than all other signs, the correct examination of the sediments is of importance to the physician.

Healthy urine forms, only after long standing, a light, sinking cloud of vesical mucus; every other separation in the urine is of a pathological nature. The urinary sediment consists either of organic formations, as mucus corpuscles, purulent corpuscles, blood, etc., or of heavy, insoluble salts or acids, or lastly, of an admixture of both; the microscope will throw light on this.

The sediment consists of organic formations. If the urine has not a blood-red color, the sediment is white, grey, dirty-yellow, and with the microscope can be seen mucus, or pus-corpuscles; here the sediment is constantly mucus, if the urine contain no albumen; it is probably pus, if the sediment is deposited rapidly after the urine is voided, and the urine contains albumen.

It is not necessary now to state of what importance it is to discover and appreciate mucus and pus in the urine; in catarrhus vesicæ the mucus sediment frequently assumes a very glutinous quality; this, however, happens only when the urine begins to become ammoniacal, which, in urine containing mucus, often occurs in a very short time, as we have already mentioned; the same may be said of pus, and it is good in this case to test the presence of albumen, not by boiling heat, but by nitric acid.

If the sediment is blood, the blood-corpuscles are then seen with the microscope; the urine standing over this is also of a blood-red color. Of the import of blood in the urine I have no remark to make.

If the urine contain albumen, and there exist at the bottom a mucus sediment, it is of great importance to examine this with the microscope. We may find therein, as I have observed, peculiar long prominences, partly filled, partly transparent, and round spheres, twice or thrice as large as mucus corpuscles, filled with dark, granular contents, which beyond a doubt have their origin in the kidneys, and denote a morbid state of this organ. These peculiar forms I have frequently, and at different times, found in the urine of a person laboring under morbus Brightii.

The sediments which are not of an organic nature, may in like manner be easily recognized with the microscope and some few reagents; they are either crystalline or amorphous, present themselves either in acid, neutral or alkaline urine, and are readily distinguished; in acid urine, sediments of uric acid present themselves, urate of ammonia, urate of soda, oxalate of lime, cystin.

The greatest number of sediments which present themselves in acid urine consist of urate of ammonia; less frequent are those consisting of uric acid; still rarer are those consisting of oxalate of lime; and the most uncommon of all are those consisting of cystin.

Sediments consisting of earthy phosphates do not occur in urine having a strong acid reaction.

Every sediment occurring in acid urine from yellow to brown, from red to purple-red, appearing under the microscope as an amorphous precipitate, or as large and small globules aggregated together, which is dissolved entirely or almost entirely on warming the urine, is urate of ammonia; to thisbelong, accordingly, all the so-called critical separations in the urine; the species of separation of the urate of ammonia is very various, and it appears sometimes as mere turbidness, without forming any sediment whatever; sometimes it lies at the bottom of the vessel as colored mucus or pus, at other times heavy, like an earthy precipitate.

In the case of those diseases, which in the course of their development admit a termination by a critical separation in the urine, the kind of separation is of importance.

The heavier the sediment lies at the bottom, and the clearer the urine is that stands over it, the more decided is the crisis allowed to be; whilst the lighter the sediment floats, and the less disposition there is to a perfect deposition, the more imperfect is Nature's effort to break down the disease by a crisis.

The various colorings of the sediment are characteristic of some diseases; in acute rheumatism of the joints, in intermittent fevers, the critical sediment is observed to be colored red up to a brown-red; in acute diseases of the liver the sediment is rose-red; in typhus it has a dirty-red color; in some diseases the appearance of the sediment appears to be of no constant critical import, as, for example, in typhus.

A sediment in the acid urine, which is not dissolved on heating the urine, and which appears crystalline when observed either by the unarmed eye, or with the microscope, and is colored yellow up to vermilion-red, is uric acid. It ordinarily appears in the form of rhombic plates, and in the majority of cases mixed with urates of ammonia, where it then forms the undermost and dark-colored layer of the sediment.

That the deposit of uric acid is of critical import, is scarcely to be doubted; in gout, and in cases of renal calculi, where the deposits consist of uric acid, uric acid or the discharge of gravel form the most perfect crisis; in many other diseases we still want the necessary observations concerning the critical value of uric acid secretions.

The sediment of oxalate of lime presents itself more rarely than those before mentioned; it usually forms a white precipitate; observed with the microscope, it appears in the form of small octahedra, or of little spheres arranged one by the other; it is not soluble in acetic acid, but readily in hydrochloric acid; when sulphuric acid is poured on it, it disappears, and after some time long lancet-formed plates of sulphate of lime are seen.

Respecting the diagnostic value of the oxalate of lime in the sediment, sufficient observations are still wanting; it is probable that it is connected with serious disturbances in the chylopoietic system; that we should be attentive to the possible formation of stone of oxalate of lime, where the sediment shows itself frequently and permanently in the urine, is of importance; however, the physician, in order to judge of the phenomena more correctly, must also have reference to the diet, as oxalic acid may be conveyed into the body by various sorts of food.

The occurrence of cystin in the urinary sediment is very rare. It is easily recognized by its remarkable form; it forms faint yellow-colored hexahedral plates.

According to Prout, the appearance of cystin in the urine is a very unfavorable sign, it indicates the formation of cystin calculi.

In neutral urine, or that with an alkaline reaction, besides the sediments already mentioned, precipitates of earthy phosphates present themselves; they are readily known by this: that they disappear on acidifying the urine with acetic acid or acid salts; the phosphate of magnesia, commonly combined with ammonia, is distinguished by its crystalline form; it appears in colorless prisms obliquely tinctured, very frequently in the form of a roof; the calcareous phosphate appears almost always as an amorphous precipitate.

As the earthy phosphates are constantly present in the normal urine, their precipitation is commonly to be looked on as a consequence of the formation of ammonia, the free acids by which the earthy phosphates were previously dissolved being neutralized by this alkali; in some cases, on the contrary, the appearance of earthy phosphates in the sediment is of diagnostic value.

In affections of the spinal cord, the phosphate of magnesia, more especially, appears to be secreted in great quantity; in affections of the mucus membrane of the bladder, the phosphate of lime appears in large quantity; in three cases of inflammations of the respiratory organs, at the time when resolution of the disease set in, I have seen the previously acid urine become neutral, and have observed, as a precipitate, the secretion of a considerable quantity of already formed crystals of ammonio-magnesian phosphate, perceptible to the naked eye, at the same time that in two of these cases the clear urine held so large a quantity of urate of ammonia in solution, that precipitates of uric acid were instantly produced by every acid.

When calculi of the bladder are present, which consist of earthy phosphates, the urine frequently contains sediments of earthy phosphates, with which a greater or smaller quantity of mucus is mixed.

In scarlatina the urine is observed to be turbid at the time of the desquamation, often also before the occurrence of the same on the outer cuticle; when it is observed with the microscope, an extraordinary great quantity of the epithelium of the vesical mucus membrane is seen in it. It is, therefore, to be admitted, that the desquamation goes on also on the mucus membrane of the bladder; and if, as frequently appears to be the case, the scaling off takes place earlier on the mucus membrane of the bladder than on the external skin, one may determine the commencement of the scaling off by examining the urine.

The knowledge of the chemical composition of the urine is of very great value for diagnosis and prognosis; especially as far as concerns the presence of matters which are not found in the normal state of the urine.

Albumen is readily discovered in the urine, by heat or by the addition of nitric acid; if the urine is acid, heat is preferred; if alkaline, nitric acid.

The presence of albumen in the urine is always of great import, and the correct estimation of the same as a means of diagnosis not altogether easy; cases are known in which albumen was observed in the urine of healthy individuals, or set in in consequence of disturbance occurring in the digestive organs; in the great majority of cases, albuminuria is the attendant of dropsical phenomena, or the forerunner of them; the urine is then commonly clear, evinces a tendency to green, and contains much albumen; but there are cases of dropsy known, which set in altogether without albuminuria.

That the presence of albumen in the urine does not infer the presence of Bright's degeneration of the kidneys, has been sufficiently proved; where degeneration of these organs is suspected, great attention must be directed to the mucus sediment of the urine.

In violent inflammations, as well as in typhus, small quantities of albumen are sometimes found in the urine; the urine is then generally very dark, and has an acid reaction. According to Becquerel, this appearance of albumen, in cases of inflammation, appears to be connected with a congested stated of the kidney, and as this, unless the disease is itself an inflammation of the kidneys, seems to occur only in very violent and intense inflammations, the appearance of albumen in the urine, accompanying inflammation, might be a sign of the intensity of the inflammation.

In consequence of inflammatory exanthems, especially in the desquamatory stage of scarlatina, albumen sometimes is observed to exist in the dark-colored urine, and sometimes, though more seldom, blood.

It is, therefore, a matter of importance for the physician carefully to examine the case, as this heterogeneous mixture is not unfrequently the forerunner of dropsy; however, dropsy has been observed after scarlatina without albumen in the urine, and albumen in the urine without dropsy following thereon.

At the commencement of diabetes mellitus, albuminuria is no rare phenomenon, and of great importance to the physician.

The occurrence of albumen in this case is not constant, but alternating, it appears, before a trace of sugar can be observed, and when the sugar begins to form, it sometimes ceases again, and passing off, it gives way to the albuminuria.

To demonstrate the presence of sugar in the urine, is the principal means of satisfying oneself of the existence of diabetes melitus. If the quantity of sugar is considerable, it is easily discovered in the alcoholic extract of the urine after evaporation; if only traces of sugar are present, the sulphate of copper is used to demonstrate its presence, as we shall see at another time.

Gall pigment in the urine is constantly a sign of the liver being affected; we have already stated that this can be discovered by the addition of nitric acid; but to infer the presence of gall pigment from the color of the urine is sometimes fallacious. *Part* viii., *p.* 49.

---

*Changes Produced in Urine by Disease.*—Dr. Shearman gives the following:

Excessive indulgence in animal food, with too little bodily exercise, dyspepsia, and want of perspiration, are always attended by increase in the quantity of uric acid and urates.

Uric acid is frequently produced in great quantity in the bladder by the hydrochloric acid formed in the stomach from disease of that organ, which, being absorbed into the blood, and secreted by the kidneys, forms hydrochlorate of ammonia, and deposits the uric acid.

In fever, and in all diseases accompanied by rapid emaciation, the urine is of a high specific gravity, a dark brown-red color from excess of urea, uric acid, urate of ammonia, and sometimes blood and purpurine.

The lateritious sediment, is urate of ammonia.

And in extreme cases of acute rheumatism and hypertrophy of the heart, very large quantities of uric acid and urate of ammonia are commonly found.

In all acute diseases attended by great emaciation, inflammation, or disorganization, with unhealthy digestive organs, as long as the kidneys remain healthy, uric acid is secreted in abundance; but if the kidneys become diseased, as in morbus Brightii, diabetes, etc., then the secretion from the kidneys is perverted, part of the nitrogen remaining in the circulation, and the carbon, hydrogen, and oxygen, assuming the forms of albumen, sugar, hippuric and oxalic acids, etc.

In diabetus melitus, when starch, sugar, etc., do not undergo the changes required to be converted into carbon or fat, the starch is converted into grape-sugar by oxygen, and the sugar is excreted by the kidneys.

In gout and rheumatism, urate of soda is found both in the urine and deposited in the joints and sheaths of the tendons.

When pressure on the renal veins exists to such an extent as to prevent the return of blood to the cavæ, as from a tumor, pregnancy, or diseased viscera, the elements of the blood are often poured out by the kidneys; and on examination, we find albumen, blood-discs, and the coloring matter of the blood (hematosine.) And in granular diseases of the kidneys, and anasarca after acute disease of the skin—as scarlitina and extensive burns—albumen in large quantities is detected, but when the kidneys and skin regain their natural functions, uric acid and urea are again secreted in the place of albumen.

In all diseases of an anæmic or chlorotic nature, attended by languid circulation and extreme debility, independent of acute disease, deficiency of urea and uric acid is found, and no deposit takes place unless there is a very small secretion of urine.

In hysteria there is a large flow of limpid urine, of low specific gravity, and of a green color.

In chlorosis, the urine is also of a low specific gravity, and green; and this green color is owing to the mixture of cystine with hemaphæin.

When the functions of the skin are impaired only, an excess of urea and urate of ammonia is always the result; and if, in this case, profuse perspiration occurs, the fluid goes off by that process instead of the kidneys.

The specific gravity of the urine becomes increased, and deposits frequently take place in the bladder in consequence, forming calculi, owing to deficiency of fluid; but, if the skin is imperspirative, then the kidneys carry off the extra quantity of water, and the urine becomes lighter; but the animal acid (lactic or butyric,) which ought to go off by the skin, is secreted by the kidneys, and combining with the ammonia or soda of the urates, produces uric acid.

When the functions of the liver are deranged, carbon is eliminated with hydrogen and cholesterine from the kidneys, which gives the peculiar color to the urine in all cases of jaundice.

In organic mischief in the liver and spleen, or great congestion of the vena portæ, the urine is very red, purple, or copper-colored, owing to purpurine and urate of ammonia; but when bile is circulating in the system from disease of the gall-ducts, etc., the urine is very brown, and easily shows the bile by the proper tests.

In contracted, hobnail, or cirrhossed liver, the extent of the disease may generally be measured by the quantity of purpurine in the urine; and usually in ascites, from diseased liver, we find purpurine: but in ascites from peritoneal disease we find none. The purpurine appears to proceed from the altered condition in the portal circulation.

When the liver and lungs are both so diseased as to prevent the proper quantity of carbon being carried off by their functions, hippuric acid is sure to be found in the urine.

In cases where organic mischief exists in the kidneys, the urine is frequently semi-solid when cold, and of a dark color, like a mass of black currant jelly.

And when hemorrhage from some part of the urinary organs takes place, the urine is red, and shows quantities of blood-discs under the microscope.

In fungus hematodes of the kidney, the urine looks like infusion of roses, while warm, and like red currant jelly when cold, taking the form of the vessel.

During the progress of pneumonia, less carbon will be eliminated from the lungs, and therefore more will be in the urine and liver; consequently hippuric acid is often found.

And in confined situations, where animals are obliged to breathe impure air, the globules of the blood are not sufficiently supplied with oxygen, carbon cannot be converted into carbonic acid in the lungs, and life could not go on unless the kidneys secreted more nitrogen and carbon in the forms of urate of ammonia and hippuric acid.

In hepatitis, the carbon is not converted into bile; and, as long as the other secretions are going on properly, the carbon which ought to form the bile is converted into fat and oil, and is found in the blood and urine.

We often meet with melancholy, highly nervous, emaciated patients, simulating diabetes mellitus, but even more depressed in spirits, who merely complain of great debility and exhaustion, with some little pain in the back or loins, for which we find it very difficult to prescribe effectually.

Dr. G. Bird has clearly shown that most of these cases are owing to imperfect assimilation in digestion, converting the urea of the nitrogenous part of the food into that state which is secreted by the kidneys, as oxalic acid and ammonia instead of sugar, which, combining with the lime of the phosphates, produces the oxalate of lime diathesis; and it is to the derangement of the stomach, duodenum, and liver, that we must look for success in the treatment of these cases.

---

*Alkaline Urine.*—When, from any cause, as the consequence of wear and tear in old age, excess of study, or great excitement of the brain, an injury to the spine, or stone in the bladder, the kidneys or bladder are deprived of their natural supply of nervous power, the elements of urea combine with the elements of water, and are converted into carbonate of ammonia, which, by irritating the mucus membrane, and neutralizing the solvent phosphoric acid, throws down the triple phosphates, and phosphate and carbonate of lime, and renders the urine alkaline.

Thus we find in almost all cases of long-continued calculi in the bladder—let the calculus be composed of what it may—that the urine is strongly alkaline, ammoniacal, and deposits phosphate of lime, which sticks to the bottom of the vessel like birdlime. The fusible calculus is composed of ammonio-phosphate of magnesia and phosphate of lime.

In persons who are confined to very sedentary habits, with great mental exertion, for a length of time, and then obliged to use violent muscular exercise for a few days, as clergymen with small livings, lawyers, and schoolmasters, alkaline urine, with abundance of earthy phosphates is usually the consequence; and this unnatural secretion brings on a degree of debility not easily accounted for in any other manner.

In irritative dyspepsia, in gouty habits, the urine often contains the phosphates in abundance; and whenever the triple phosphates, with alkaline urine, are deposited for a length of time together, both in the night and morning urine, accompanied by emaciation, there will always be found organic disease, either in the digestive organs, kidneys, or bladder, if not in the spine.

Dr. Golding Bird gives the following rule respecting the phosphates, which will be found very useful in practice: That where the presence of phosphates is only found in the evening urine, organic disease is rarely the cause of it; but where they are found equally in the morning and evening urine, you may be sure organic disease exists.

In bad cases of typhus fever, the urine is frequently ammoniacal toward the close of the disease, the nervous system of the kidneys being too depressed to secrete urea, and its elements being converted into carbonate of ammonia, just as they would be in common chemical decomposition out of the body.

During retention of urine from diseased prostrate gland, stricture of the urethra, or where a catheter is obliged to be worn, the urine is always alkaline, owing to irritation in the mucus membrane.

It is easy, after a few trials, to discovor, in a few minutes, the contents of almost any ordinary specimen of urine; and, without this knowledge, it is quite impossible to become acquainted with the changes which are constantly occurring in the urinary organs.

For instance, you are called to a patient complaining of excruciating and deep-seated pain in the abdomen, shooting down the thigh, which resists the usual soothing mode of treatment. Examine the urine, and you may there find blood, pus, oxalate of lime, uric acid, or phosphates. This at once explains the nature of the malady, and you can confidently tell your patient that he has a small calculus of a certain description in the ureter, and your treatment is no longer empirical.

Or you may be consulted by a person with various anomalous symptoms of a cachectic nature, which you would find it impossible to give a name to unless you examine the urine chemically, when you discover either albumen, sugar, or oxalate of lime excreted; and this at once explains the case.

A patient consults you, much reduced in strength, depressed in spirits, and emaciated, his appetite being good, and digestive organs being healthy, as far as you can discover, there being no evident cause for such a state. Take the specific gravity and quantity of the urine. If it is high, without deposit, and the quantity not great, add nitric acid to it in a watch-glass, and you will most likely discover a large quantity of urea, sufficient at once to account for the symptoms; or if the quantity of urine be large, you may discover sugar.

During the course of febrile and inflammatory diseases, urate of ammonia is generally deposited. But if the symptoms increase, instead of diminish, under this deposit, examine it, and perhaps you may find the deposit to consist of phosphates and purpurine, and the urine alkaline. This will at once warn you to be watchful of your patient, and cautious in your prognosis.

Suppose you have a surgical operation to perform on a patient apparently out of health, but with no decided disease. If, on examining the urine, you find albumen, oxalate of lime, or the ammonio-phosphates of magnesia in abuudance, you may feel assured the person is in an unfit state to bear any operation; and by waiting, and attending to his general health, you will feel more confidence in the successful event of such an operation. *Part* xii., *p.* 96.

*Test for Sugar in Urine.*—Lowig recommends the following: Evaporate the urine to the consistency of syrup; treat with alcohol; then to the solution add an alcoholic solution of potash; if sugar be present, a white precipitate is formed, consisting of a compound of sugar and potash; wash this with alcohol, and dissolve in water; thus a saccharine solution is formed, which may be examined in any way, and if required, the amount of sugar determined quantitatively.
*Part* xii., *p.* 117.

*Alkaline Urine.*—Use strichnia when the affection follows injury or lesion of the spine, as recommended by Dr. Golding Bird. *Part* xiv., *p.* 97.

*Influence of the Rhubarb Plant in producing Oxalate of Lime in Urine.*—[The oxalate of lime in urine, can only be considered diagnostic of disease when the oxalic acid is known to have been generated within the economy, and not introduced from withont, for, says Liebig, the analysis of urine, when made without respect to the inorganic salts, acids, and bases of the aliment, teaches nothing. Are there any kinds of food which may be the means of impregnating the urine with oxalate of lime? Dr. Wilson says:]

The young stocks of the rhubarb plant, which of late years have come into such general use in this country, for tarts in the spring, and sorrel, of which our neighbors, the French, consume a good deal in salads, and in other ways, both contain oxalic acid; and hard water contains lime. Dyspeptic persons who drink such water, and eat such articles of food, and are thus daily introducing, without suspecting it, the constituent ingredients of the mulberry calculus, are very likely indeed to incur the pain and the exceeding peril of a renal concretion of that kind. You must see, therefore, the great importance of detecting the oxalic diathesis, and of forbidding, to those who have it, all such viands as contain the oxalic acid, and of recommending them to use pure water, even distilled water for drinking.
*Part* xiv., *p.* 108.

*To Render the Urine Acid.*—Dr. Bird states that the benzoic is the only acid, the exhibition of which will render the urine acid. *Part* xv., *p.* 143.

*Alkaline Urine.*—Wash the bladder out every day or two with warm water; the urine is rendered alkaline by retention of a few drops in the bladder. But Dr. Snow considers it important to distinguish those cases in which the urine is *secreted* alkaline. *Part* xv., *p.* 144.

*Incontinence of Urine in Children.*—[In the incontinence of urine occurring during sleep, in children, and unconnected with organic disease, Dr. Chambers advises the following treatment:]

Let the patient abstain from drinks for three hours before bed-time; empty the bladder on going to bed, and awaken in three hours to make water again. Blister the sacrum occasionally, to prevent sleeping on the back, and to stimulate the bladder; use cold shower bath or douche, and give ten drops each of tinct. cantharid. and tinct. ferri mur. thrice a day. Sometimes cauterize the orifice of the urethra, that the passage of urine may excite pain. *Part* xv., *p.* 145.

*Incontinence of Urine in Young Persons.*—M. Gerdy advises to give strychnine, gr. $\frac{1}{4}$ to $\frac{1}{2}$ daily, and small enemata of quinæ sulph.; or give ext. belladon. gr. iij. daily. Continue the remedies for some time after it is apparently cured.

When the urine is catarrhal, inject into the bladder a solution of nitrate of silver; give ergot of rye and camphor, aa. gr. x. daily, and rub the perineum and pelvis with camphor ointment. Or give strychnine or quinine, or four grs. of cantharides powder daily. *Part* xv., *p.* 146.

